# Putative biotic drivers of plant phenology: With special reference to pathogens and deciduousness

**DOI:** 10.1002/ece3.8932

**Published:** 2022-06-02

**Authors:** Rowland D. Burdon, Michael J. Bartlett

**Affiliations:** ^1^ Scion (New Zealand Forest Research Institute Ltd) Rotorua New Zealand

**Keywords:** biotic interactions, deciduous, evolution, herbivory, pathogens, plant phenology

## Abstract

Plant phenology is not only manifested in the seasonal timing of vegetative and reproductive processes but also has ontogenetic aspects. The adaptive basis of seasonal phenology has been considered mainly in terms of climatic drivers. However, some biotic factors as likely evolutionary influences on plants’ phenology appear to have been under‐researched. Several specific cases of putative biotic factors driving plant phenology are outlined, involving both herbivores and pathogens. These illustrate the diversity of likely interactions rather than any systematic coverage or review. Emphasis is on woody perennials, in which phenology is often most multifaceted and complicated by the ontogenetic aspect. The complete seasonal leaf fall that characterizes deciduous plants may be a very important defense against some pathogens. Whether biotic influences drive acquisition or long‐term persistence of deciduousness is considered. In one case, of leaf rusts in poplars, countervailing influences of the rusts and climate suggest persistence. Often, however, biotic and environmental influences likely reinforce each other. The timing and duration of shoot flushing may in at least some cases contribute to defenses against herbivores, largely through brief periods of “predator satiation” when plant tissues have highest food value. Wide re‐examination of plant phenology, accommodating the roles of biotic factors and their interplays with environments as additional adaptive drivers, is advocated toward developing and applying hypotheses that are observationally or experimentally testable.

## Graphical abstract

It is postulated that not only biotic factors, especially pathogens, but also herbivores have played an under‐rated role in the evolution of plant seasonal ecology. A selection of case histories is reviewed, a classic case being deciduousness in the poplar rust pathosystem.

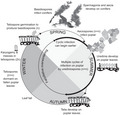

## 1 INTRODUCTION

The phenology of an organism is among the key bases of evolutionary adaptation to its habitat. It involves timing of an array of developmental processes, with seasonal and ontogenetic aspects. With plants at least, phenology is generally addressed in terms of its seasonal aspect, which we mainly address. Seasonal phenology involves seasonal timing of growth and reproductive processes. The visible processes in plants include bud break in spring, shoot elongation, bud set in autumn, natural foliage shedding, production of flower (or strobilus in gymnosperms) buds, actual flowering, and development of fruit and eventual fruit drop or seed shedding. Phenology, however, can also include cryptic features (cf. Albert et al., 2019). These include initiation and differentiation of primordia, seed development, timing of cambial activity, dehardening and hardening of tissues against frost, the nature of growth rings, and seasonal patterns in root development. However, we will not address specifically the cryptic phenomena, which include both precursors of and flow‐on from the visible ones. In any event, the fullest, most multifaceted expression of phenology tends to be in woody perennials, which feature predominantly in this article.

The evolutionary drivers of phenology are of more than purely scientific interest. They are also of increasing interest with the accentuated adaptive pressures on plant populations imposed by climate change (e.g., Kijowska‐Oberc et al., 2020), ecosystem fragmentation, and the arrival of new pests and pathogens. Understanding both the evolutionary drivers and the increased pressures will help in future management decisions. An extensive literature on evolutionary drivers of phenology has focused mainly on climates, with their seasonal cycles, the topic being complicated by extreme diversity of adaptive strategies. Numerous trade‐offs arise between different features of strategies that may contribute to fitness in particular environments. Adaptive strategies for any organisms are thus highly multidimensional, being characterized by how different features interact with each other to create ecological fitness, and not by any single features. Even within a single habitat, different plant species typically vary widely in their adaptive strategies. This is obvious for co‐occurring species that are evergreen or deciduous as the case may be. Less obvious, though, are some specific selective forces that shape individual strategies.

Ontogenetic phenology is considered briefly toward the end of this article to give perspective. It centers on how plants, as they get older and larger, undergo maturation, which is manifested in widely varying degrees (Poethig, 1990; Zotz et al., 2011). It generally involves acquisition of reproductive competence, which introduces a whole suite of seasonal expression. Even vegetatively, there can be a transition from a juvenile habit to a mature one, or in some species a relatively abrupt switch (Zotz et al., 2011). Whatever the case, there can be major changes in gross morphology and tissue anatomy.

Some consideration has been given to the interplays between biotic factors and plant phenology. Both biotic and abiotic selective influences were considered decades ago in reviews focused on plant phenology. Rathcke and Lacey (1985), however, focused heavily on quantitative descriptors of phenology, namely timing, duration, synchrony, and statistical distributions of phenological events. Van Schaik et al. (1993) emphasized seasonal climatic drivers of phenology, with the implications for herbivores (“consumers”). Recently, in relation to the relative importance of biotic drivers of local adaptation, meta‐analyses have been conducted—albeit with no specific focus on phenology. Hargreaves et al. (2020) did so for biotic effects in plants only, and Briscoe Runquist et al. (2020) for abiotic and biotic effects in plants and animals. Some trends were detected, notably in biotic influences being more important in tropical than in temperate latitudes. However, results were very heterogeneous and perennial plants were weakly represented. One area that has been significantly researched is seasonal phenology of leaf production in relation to herbivory within the tropics (Lamarre et al., 2014). In some other cases, the research focus has been on the seasonal phenology of herbivorous insects adjusting to that of the host plant (Chuine, 2010; Elzinga et al., 2007; Singer & Parmesan, 2010). In a context of mammalian herbivory, Benning et al. (2018) cite a case of a plant's reproductive phenology constraining its geographic range.

Despite these considerations, the possible extent and diversity of putative biotic factors as evolutionary drivers of seasonal plant phenology appear to remain under‐researched, especially with pathogens. Such factors represent the main focus of this study. We consider the evolutionary importance of biotic interactions, involving pathogens and herbivory, with plant phenology. Main emphasis is on seasonal phenology, with brief consideration of ontogenetic phenology. While some postulated cases are cited from the literature, we offer others. Among postulated cases, involving both pathogens and herbivores, we first consider briefly the timing and synchronicity of both vegetative growth (bud burst, shoot flushing, and bud set) and flowering. We then focus strongly on the complete seasonal leaf fall that characterizes the deciduous habit, in relation to selective pressures imposed by pathogens and herbivores, and address the evolutionary hurdles facing shifts between the deciduous and evergreen habits. Several other cases of putative biotic drivers are also outlined. Coverage involves mainly woody perennial plants, but two cases with pasture plants are considered, along with one involving various wild herbaceous plants. As such, our coverage serves to illustrate the diversity of likely biotic interactions involving plant phenology, and the need for additional investigation, rather than any attempt at comprehensive or even systematic review. We draw heavily on close research familiarity with certain species, hoping that the value of some detailed case histories outweighs the sparseness of the coverage.

In considering the evolutionary importance of biotic interactions, involving pathogens and herbivory, with plant phenology, our main emphasis is on seasonal phenology. Ontogenetic phenology is considered briefly.

## 2 SEASONAL PHENOLOGY

We note initially some key features of seasonal phenology, in relation to cues and selective forces involving climate, as background for considering interactions with pathogens and herbivory.

Traits of seasonal phenology are often both variable and heritable within populations (Li & Adams, 1993; Matziris, 1994; Skrøppa & Steffenrem, 2019), giving scope for evolutionary change. Nevertheless, limits to a species’ phenology can still govern its geographic range (Chuine, 2010), which of course may be altered by climatic change. However, our focus is on likely drivers of the phenology of populations wherever they are growing, considering vegetative and reproductive aspects separately.

### 2.1 Vegetative aspect

In the seasonal timing of vegetative shoot phenology, there are the obvious climatic drivers, reviewed in detail by Axelrod (1966). A key feature is timing of bud burst, or flushing, which typically occurs in spring in temperate climates or in some relation to wet and dry seasons in tropical or semi‐tropical climates. Also typical is a close seasonal concentration and synchronization of flushing. In temperate or cool temperate climates, optimal fitness will classically represent some balance between the advantages of flushing as soon as conditions favor growth and the safety of delaying it until after almost all threat from late (spring) frost. The period of shoot extension growth that follows flushing can be very brief, certainly in many temperate tree species, including various conifers. Often these seasons seem far shorter than the periods when climatic conditions would permit active growth, classic examples existing among the true firs (*Abies* spp.). Complementing flushing and subsequent shoot extension are bud set associated with the cessation of elongation, and the complete shedding of foliage in deciduous species. These processes occur in late summer or autumn in temperate or cool temperate climates, but in variable relationships to dry seasons at lower latitudes.

### 2.2 Reproductive aspect

Seasonal synchronization of flowering is often closely linked to vegetative phenology. This has several obvious benefits, apart from those incidental to optimal timing with respect to climatic factors. Among the benefits, close synchronization helps assure adequate pollen when the female structures are receptive. Also, by all occurring early enough, this assures sufficient time for fruits and seeds to develop and mature before either summer drought or autumn cold disrupt such processes. Moreover, brief but differentiated flowering seasons can also provide reproductive barriers between species that are interfertile but whose hybrids may not be ecologically fit; however, such barriers have broken down in some eucalypt species grown as exotics where their flowering seasons differ from those in native habitats (Eldridge et al., 1993). This synchrony can mean very seasonal food supplies for animal pollinators, but very short seasons can be made good by other sympatric species having differentiated flowering seasons—as with various eucalypt species (Eldridge et al., 1993). A further but probably very incidental benefit of flowering synchrony may be creating a brief superabundance for herbivores, which we will consider later.

Masting, namely heavy flowering and seed production in some years, with little or none in between (Kelly, 1994; Kelly & Sork, 2002), is a phenomenon involving the timing of flowering. Its postulated biotic significance is as a means of alternating between occasional predator satiation and more frequent predator avoidance. However, it is not actually a seasonal phenomenon, or really ontogenetic, so it is not considered further.

Timing of flowering has been reported as a minor factor in complex interactions involving transmission of pathogens by pollinators (Biere & Antonovics, 1996; McArt et al., 2014), but is not considered further in this connection.

### 2.3 Pathogens as putative drivers

The role of pathogens as potential evolutionary drivers of seasonal phenology appears to have been especially under‐researched. Cases we consider involve: potential roles of pathogens in governing the deciduous growth habit; the seasonal timing of spring flushing in a widely distributed conifer; two closely related *Pinus* species with overlapping distributions but markedly different seasonal timing of vegetative and reproductive processes; and an oak species in what is effectively an altitudinal transect. Briefly considered also is how pathogens can modify phenotypically some processes that are normally parts of seasonal phenology.

#### 2.3.1 Deciduous versus evergreen habit

Of special note in phenology is the seasonal complete shedding of foliage that characterizes the deciduous habit, versus the evergreen habit. This was reviewed by Holttum (1953) for tropical forests and Axelrod (1966) for temperate forests. These important papers pointed to factors of the abiotic environment as the evolutionary drivers of deciduousness. This focus on abiotic factors appears to have dominated subsequent enquiry into the topic. Both authors postulated that seasonal drought was the original driver. Specifically, Axelrod postulated that this habit, having originated during the Cretaceous, was a preadaptation to cold winters that subsequently characterized high‐latitude environments along with their widely fluctuating day lengths. Yet, in many harsh, high‐latitude climates, with very severe winters, both deciduous and evergreen tree species co‐occur. Moreover, even within some genera, notably *Nothofagus* in South America, both deciduous and evergreen species can grow adjacently. In adaptation to climates with severe winters, there are some obvious trade‐offs. The deciduous habit entails heavy seasonal turnover of biomass, but the leaves do not require investment in anatomical features needed for overwintering. This habit also allows the leaves to be photosynthetically very efficient relative to their dry matter (Reich et al., 1992). Apart from co‐occurring species, sometimes close relatives, including both evergreens and deciduous ones, there are other obstacles to facile climatic interpretation. For instance, deciduousness exists among many species without severe winters (Li et al., 2013; Suc, 1984). Also, there are deciduous tropical tree species that produce new foliage well before dry seasons end, which we will revisit.

For the evergreen habit, the anatomical requirements for leaves surviving winters or other seasonal stresses will also tend to make them less attractive or rewarding to herbivores in general. Such protection against herbivory is very often complemented by toxin production. All these defenses, along with defenses against abiotic factors, require additional investments (Kursar & Coley, 2003; Loehle & Namkoong, 1987; Strauss et al., 2002; Villar et al., 2006), or “higher construction costs” (Smith et al., 2019). However, such investments can obviate the cost of the complete seasonal turnover of foliage in deciduous plants. Also, early flushing is not crucial for evergreens to resume photosynthesis in the spring (cf. Panchen et al., 2014). Osada (2020), comparing sympatric evergreen and deciduous species, found the former to have later and longer periods of leaf expansion. One might expect foliage toxicity to be more prevalent and more severe in evergreen species than in deciduous ones, but we have found no published survey on this question. Anecdotally, however, yews (*Taxus* spp.) which are evergreen are both notoriously toxic and often associates of deciduous species. Within a deciduous example (*Populus deltoides* × *P*. *nigra “Robusta”*), a subtle, seasonal effect of seasonal decline in phenolic compounds being associated with increasing susceptibility to the leaf rust *Melampsora larici*‐*populina* Kleb. was observed by Maupetit et al. (2018). This suggests that, if phenolic compounds represent a defense mechanism, that decline reflects an energetic cost of their production and decreasing value of the protection that they confer.

While much attention have been given to the energetic costs and associated trade‐offs in alternative strategies of deciduous or evergreen habit, little seems to have been given to the possible roles of pathogens and herbivores in generating or maintaining the deciduous habit. In relation to the evolutionary pressures imposed by both biotic and abiotic factors, we will consider also the evolutionary hurdles to be overcome in shifting from one habit to the other. Regarding deciduousness, the presence and life cycles of pathogens may play a role, and we now consider a probable example.

##### The poplar rust case

**FIGURE 1:**
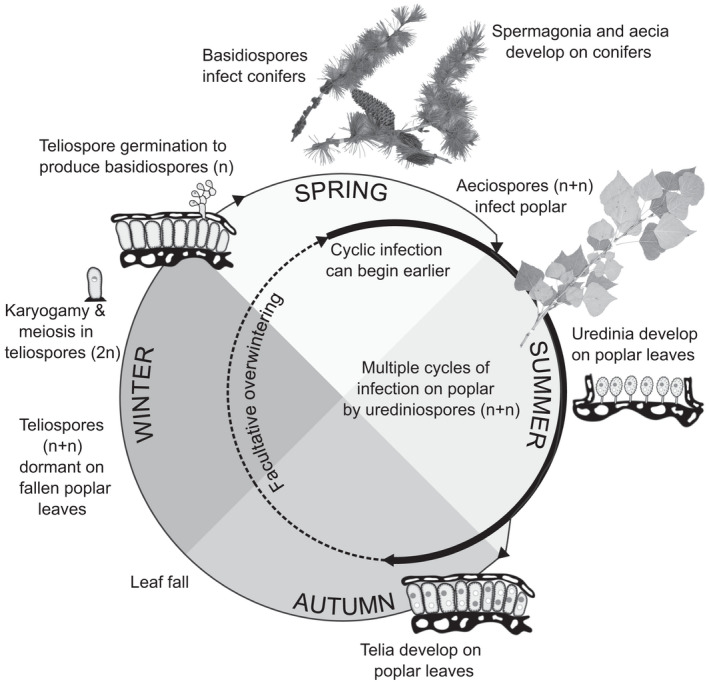
Life cycle of poplar rusts in New Zealand. The smaller inner circle represents polycyclic infection of poplars by urediniospores, with the potential epidemic period represented by the bold line, and potential for facultative overwintering on poplar is represented by the dashed line. Continued cycling by *Melampsora larici*‐*populina* during mild winters on semi‐evergreen poplars facilitates earlier build‐up of epidemics. *Melampsora medusea* can only infect poplar and must overwinter in the uredinial stage, possibly via mycelia surviving in leaf buds. The larger outer circle illustrates the full macrocyclic life cycle of *M*. *larici*‐*populina* involving four additional spore stages in which the pathogen must infect the alternate host (conifers, primarily larch (*Larix* sp.)) before the aeciospore stage can infect poplar and resume the cyclic urediniospore stage. This figure is based on figures presented in Spiers (1990) and Vialle et al. (2011). Images of hosts were sourced from the National Forestry Herbarium, Scion. 2020. NZFRI online dataset, nzfri.scionresearch.com, accessed in March 2022

A clue came when two poplar (*Populus*) leaf rusts, caused by *Melampsora medusae* Thum. and *M*. *larici*‐*populina*, reached Australia and hence New Zealand in 1973 (Spiers, 1998; Van Krayenoord et al., 1974; Wilkinson & Spiers, 1976). These fungal pathogens, between them, severely affected most of the planted poplar clones in both countries. While *M*. *medusae* can only affect poplar, *M*. *larici*‐*populina* has a potentially complex life cycle, which in its most complex form is illustrated in Figure 1. While the poplar hosts are in leaf, both these rusts spread by urediniospores (asexual spore stage), this spread being polycyclic with repeated cycles of infection within the season. At the start of the autumn, thick‐walled, dikaryotic teliospores are produced by *M*. *larici*‐*populina*, which overwinter on fallen poplar leaves and begin the sexual stage of the life cycle. Teliospores germinate in spring, producing basidiospores that infect the alternate, conifer host, generally larch (*Larix* sp.). Infection of poplar from mid‐spring begins from aeciospores, produced on the conifer. This completes the sexual stage of the life cycle, starting afresh the polycyclic seasonal build‐up of infection. Where the conifer host is not present, however, some overwintering of the uredinial stage, either on persisting poplar leaves in mild climates or possibly as mycelia within dormant leaf buds, can start the build‐up (Albornoz et al., 2018; Barrès et al., 2012; Walker et al., 1974; Wilkinson & Spiers, 1976). In a co‐evolved host/pathogen relationship involving such leaf rusts, there should be a comparatively slow build‐up of infection during the growing season. However, overwintering cycles of urediniospore production would accelerate the new seasonal build‐up. In New Zealand, a semi‐evergreen mutant clone (cv. Sempervirens) of the Lombardy poplar (*Populus nigra* L. cv. Italica) had been widely planted, being often favored for orchard shelterbelts. This had originated in Chile as a somatic mutation. Its widespread presence, with the semi‐evergreen habit and the inherent susceptibility to *M*. *larici*‐*populina* (but not to *M*. *medusae*), exacerbated the initial rust epidemic. Where winter conditions are suitably mild to allow retention of leaves, the all‐year presence of infected leaves assured a continuing supply of urediniospores, favoring a far quicker build‐up of infection in the new season than would be expected from deciduous material. Aided by this quick build‐up, *M*. *larici*‐*populina* spread rapidly and widely, defoliating poplars susceptible to both rusts, while *M*. *medusae* unable to build‐up inoculum in the winter was outcompeted and remained restricted in distribution (Wilkinson & Spiers, 1976). The semi‐evergreen clone was very severely affected, and was soon almost eradicated, which presumably contributed to the rusts becoming less serious a few years after arriving. Other contributing factors would have included felling of trees of other severely affected cultivars, and a spontaneous proliferation of hyperparasitic and otherwise antagonistic microorganisms in phyllosphere communities on the leaf surfaces (Heather & Chandrashekar, 1982).

**FIGURE 2:**
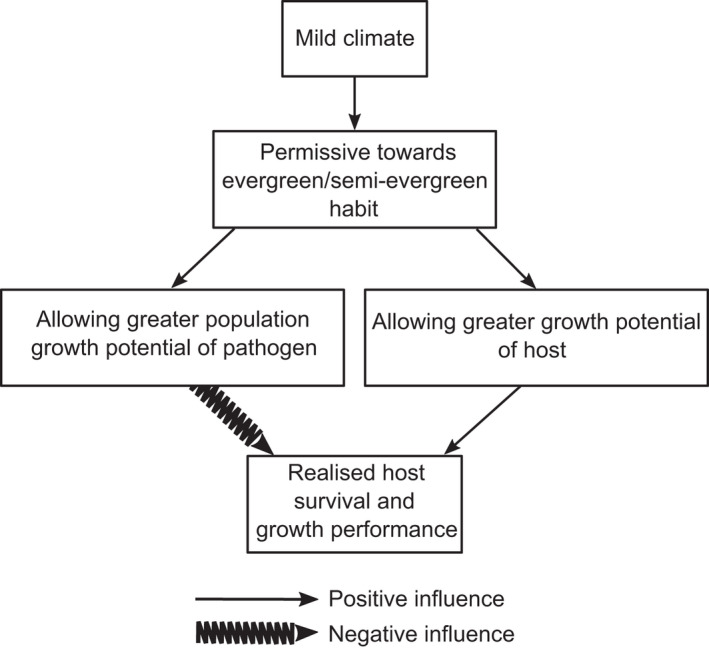
Flow chart showing expected countervailing influences of mild climate with semi‐evergreen habit in poplar rust pathosystem

The semi‐evergreen cultivar shared the inherent susceptibility to infection with the original deciduous cultivar. That susceptibility, however, has exposed a potential danger of an evergreen habit in reducing protection from natural delays in the seasonal build‐up of infection. Such a reduction can be expected to increase the level of resistance required for field fitness which would increase selection pressures imposed by rusts that operate alongside all the other selection pressures. Since increased selection pressure on any trait tends to reduce the possible selection intensity for other traits, this means a potential for “selection overload.” Moreover, the poplar rust pathosystems are very complex (Albornoz et al., 2018; Barrès et al., 2012; Persoons et al., 2014), with a potential to be very dynamic and readily destabilized. Table 1 summarizes the expected impacts of rust on the poplar host, in scenarios varying according to: severity of winter, presence or absence of alternate (conifer) hosts, and deciduousness or an evergreen habit. Figure 2 outlines expected functional interplays, under a mild climate, between evergreen/semi‐evergreen habit, growth potential, infection level, and host performance.

**TABLE 1:** Expected impacts of alternative poplar rust scenarios on poplar host (see *The poplar rust case*)

Nature of winters, Poplar habit	Alternate (conifer) host	Overwintering mode for rust	Spring base level of inoculum	Rate of seasonal build‐up of inoculum	Impact on host
Cool to cold Poplar deciduous	Present	Teliospores	Very low	Very slow	Mild
Cool to cold Poplar deciduous	Absent	Urediniospore survival (facultative)	Very low	Slow to very slow	Mild
Mild Poplar deciduous	Present or absent	Urediniospores or teliospores	Low	Slow	Mild
Mild, poplar evergreen or semi‐evergreen	Immaterial	Continued production of urediniospores	High	Very rapid	Very adverse

Co‐adaptation between rusts and poplars has tended to arise in climates that have severe or moderately severe winters. Thus, the mild climates of New Zealand and Chile are not the norm for the natural pathosystems. Yet, these novel conditions outside climatic norms provide an opportunity to examine the action of pathogens as evolutionary drivers influencing the persistence of deciduousness. Testing our hypothesis, and whether an evergreen poplar could ever be ecologically fit, however, faces major practical obstacles. Ideally, a population of evergreen genotypes, rather than just one, should be used for comparison with deciduous genotypes. However, there is no easy pathway to obtaining such a population. Even using the existing semi‐evergreen clone would be suspect because it might not match young seedlings, from which native poplars grow, for susceptibility to the pathogen within the growing season. Indeed, any matching of comparison material for inherent susceptibility would be challenging. Field testing would be wanted, where natural pathogens would be present. The impact of the pathogen(s) would need to be evaluated, most likely over several years as most impacts from rusts occur after repeated seasons of infection. Evaluating impact is likely to be complicated by the fact that presence of a susceptible evergreen host will likely influence disease development and impact on deciduous genotypes. Pathogen dispersal between stands could similarly complicate the picture. To manage these challenges, chemical control (or some effective but targeted biocontrol) of the pathogen(s) would need to be used to exclude rust impacts from a subset of trees. Support for our hypothesis would be obtained from the persistence of susceptible evergreen genotypes, and the relative success compared to susceptible deciduous genotypes, where the pathogen impacts are excluded from both, and the decline and eventual extinction of evergreen genotypes where pathogen impacts are not prevented.

##### Some deciduous tropical trees

Some deciduous tropical tree species behave very counterintuitively in producing fresh foliage before a dry season ends (Borchert & Rivera, 2001; Frankie et al., 1974; Kushwaha et al., 2015). A specific case is *Faidherbia albida* A. Chev. (apple‐ring acacia), a widely distributed, deep‐rooting tropical tree species of Africa, found with annual rainfall of 200−500 mm. It flushes vegetatively during the dry season, but sheds its leaves during the rains (Huxley, 2001), yet it flowers at the end of the wet season. Accounting for such counterintuitive behavior is a challenge, but it is hard to see a convincing alternative to the postulate of pathogens driving a shift to deciduousness. Actual support for the postulate would come if, when such species are grown with no dry season, the new foliage suffered from pathogens. The success of such a study, however, would depend on both the presence of the appropriate pathogens and the nature of the triggers for shedding old leaves and producing new ones. An alternative driver, namely increased herbivory pressure during the wet season, seems very unlikely in these cases.

Suggesting a similar explanation are some results of Frankie et al. (1974), for “Wet” forests in Costa Rica, where dry seasons would not be severe and not generally associated with actual deciduousness. They noted “Most Wet forest species flushed large quantities of new leaves during the first dry season.”

##### Evolutionary barriers to switches of habit

The *Melampsora* story raises two questions: (1) whether the influence of pathogens serves to maintain a habit, or drives switches from one to the other; and (2) if the latter, in what direction? It was long ago proposed (Axelrod, 1966; Holttum, 1953) that the deciduous habit arose as a defense against seasonal stresses. But, once established, it may persist, in a state of “biotic lock‐in,” due to factors like the behavior of *Melampsora* rusts. Answers to both questions, however, depend on the evolutionary hurdles facing the respective switches.

The evolutionary hurdles for a shift in habit will differ between the directions of switch. A related question is what factors, in what combinations, may either drive a switch or prevent one? Another question involves relationships between individual fitness and population fitness. An evolutionary switch from a deciduous to an evergreen habit might be expected to be favored by a mild climate, imposing no stringent requirements for anatomical and physiological adjustments to abiotic factors. However, it would likely require adjustment to biotic factors. These might include enhanced morphological, anatomical, or chemical defenses against herbivores, or evolution of greater pathogen resistance. A mild climate, if humid, tends to favor various fungal or oomycete pathogens, creating a need for greater resistance. Thus, an evolutionary inertia may work against a switch to an evergreen habit. A potential corollary to that is restriction of the host's geographic range to where deciduousness does not incur a decisive competitive disadvantage.

In a co‐adapted relationship between a temperate‐zone evergreen host and a pathogen, the likely gains from becoming deciduous in avoiding infection pressure appear minor, especially compared with the selective and energetic costs of acquiring the habit. With a new pathogen arriving, the comparative fitness advantages and selective costs can change radically, as with *Melampsora*. This risk is much elevated by increasing human influences, from long‐distance trade and travel.

##### Discussion

**FIGURE 3:**
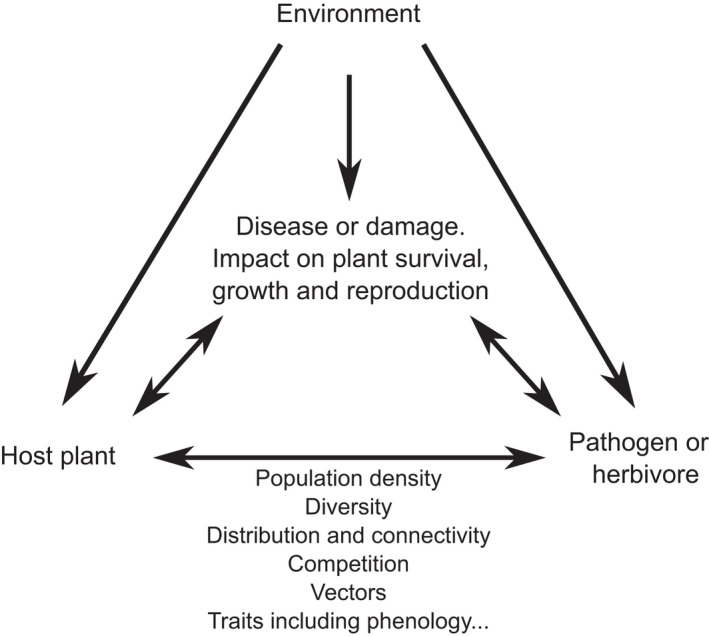
Diagram outlining interplays between factors in the “triangle” involving host plant, pathogen, and environment

We are not postulating that pathogens have necessarily created a biotic lock‐in of deciduousness, but rather that the evolutionary inertia may have been decisive in some cases but not others. The evolutionary outcome could likely be influenced by various factors, including details of the pathogen life cycle. Possibly relevant details include: longevity of fungal fruiting bodies, level of host specificity, and absence or presence of alternate hosts. If alternate hosts exist, whether they are obligate, and what phases of the pathogen life occur in which host, are also likely to be relevant. A useful framework to examine these complex co‐evolutionary dynamics that includes the context of abiotic factors is the “disease triangle” (Stevens, 1960), which illustrates the interplay among host, pathogen, and environment, and their respective interaction and influence on disease and impact upon the host (Figure 3).

The story for tropical tree species does appear to be different. The production of foliage during the dry season in some species suggests strongly that, if pathogens are a selective force, they would have sometimes driven a switch to deciduousness.

The picture concerning the general incidence of deciduousness among woody perennial taxa is mixed. Conifers, which represent the ancient taxon, are very predominantly evergreen, suggesting profound evolutionary inertia against a shift to deciduousness. One exception is the entire genus *Larix*, within the Pinaceae, being deciduous. The remaining exceptions are all Taxodiaceae members of the Cupressaeae/Taxodiaceae complex, namely the genera *Taxodium*, *Glyptostrobus*, and *Metasequoia*, although *T*. *mucronatum* Ten. is semi‐deciduous. They also tend to have few pathogens, along with some other taxonomically isolated species. Among angiosperms, which are evolutionarily more recent, deciduousness is far more common in numbers of species if not in percent of total species. Even with genera, sympatric species can include both deciduous and evergreen members. This is so with *Nothofagus* in South America (as already mentioned), and *Quercus* in North America (where some species are semi‐evergreen/deciduous) and the Mediterranean basin. Overall, the general incidence of deciduousness among taxa argues against any completely overriding influence of abiotic environment or taxonomic lineage *per se*.

#### 2.3.2 Swiss needle cast and Douglas fir

With native populations of Douglas fir (*Pseudotsuga menziesii* (Mirb.) Franco in western North America, dates of spring bud burst show a coast‐to‐inland gradient from the Pacific coast. At a given elevation, bud burst comes earlier the further from the coast (Campbell & Sugano, 1979). This has been observed not only *in situ* but also in common garden experiments, ruling out a simple effect of cooler coastal temperatures caused by the cold ocean current. One possible reason is that coastal populations have a longer humid season in which to complete vegetative growth and cone ripening, especially compared with more easterly populations where summer drought starts earlier (cf. Campbell & Sorensen, 1978). Another possible factor may be less insolation early in spring. Both factors might reduce the advantages of early bud burst in coastal populations. Yet, another possibility, not mutually exclusive, is that later bud burst there escapes the worst of the seasonal hazard, created by humidity, of infection by foliage pathogens. Of such pathogens, the most prominent is *Phaeocryptopus gaeumannii* (T. Rohde) Petrak, cause of Swiss needle cast (Boyce, 1940; Mulvey et al., 2013), which is most aggressive in the humid coastal climates. Given the postulated selection pressure imposed by the pathogen, one might expect fog‐belt Douglas fir grown in very mild climates without the pathogen to show a genetic shift to earlier bud burst. However, any such study would both be long term and face the difficulty of finding anywhere that will remain free of the pathogen. Chemical control of the pathogen would need to be long term and is expensive, and could be complicated by the pathogen evolving resistance.

#### 2.3.3 Two *Pinus* species

Two closely related coastal California pine species appear to be another case in point. They are *Pinus radiat*a D. Don (either Monterey pine or radiata pine) and *P*. *muricata* D. Don (bishop pine or muricata pine). They have overlapping (but almost entirely allopatric) geographic distributions. *Pinus radiata* ranges discontinuously from 28−37°N, and *P*. *muricata* discontinuously from 31^1^/_2_−41°N. *Pinus radiata* has a very long, opportunistic growing season, being able to make growth all year if temperatures permit, which evidently accounts for a very fast growth potential (Burdon, 2001; Burdon et al., 2017). By comparison, *P*. *muricata* makes minimal shoot elongation during winter, with apical buds remaining sealed, and is slower growing. Correspondingly, it has a later pollination season. These species are naturally challenged by strongly overlapping sets of pathogens, notably ones causing needle casts and shoot galls.

##### Needle casts

*Pinus radiata*, when grown in the British Isles, is very subject to a foliage disorder called “yellows” (Fennessy et al., 2012; Lally & Thompson, 2000). This is very severe needle cast, which was eventually linked to the fungus *Cyclaneusma minus* (Butin) DiCosmo after problems with false‐negative isolations. The disease occurs after long spells of mild, cloudy weather in winter and early spring which mean very low insolation at such latitudes. Similar needle casts have occurred in New Zealand, associated with prolonged very wet and mild winter weather. One of these has been attributed to *C*. *minus* emerging from latent pathogen status to cause “spring needle cast.” Another, called “physiological needle blight,” has been unexplained but is now suspected to have been caused by the oomycete *Phytophthora kernoviae* Brasier, Beales & S.A. Kirk (McDougal et al., 2021). Yet another, “red needle cast,” has been associated mainly with *Phytophthora pluvialis* Reeser, Sutton & Hansen, and to a lesser extent with *P*. *kernoviae* (Fraser et al., 2020). Such weather, with temperatures allowing growth but with low insolation restricting photosynthesis, is likely to deplete the carbohydrate reserves that would help the host resist pathogens. By comparison, the more winter‐dormant Guadalupe Island provenance of *Pinus radiata* is less prone to yellows (Fennessy et al., 2012) than cultivated *P*. *radiata* stocks of mainland California origin. Also, northern populations (Lat. >38°N in California) of *P*. *muricata*, which is much more winter dormant, are far less subject to such needle casts. While this comparison involves both species as exotics, rather than in natural habitats, it suggests that pathogen pressure can favor winter quiescence despite winter temperatures that permit active growth. That said, *P*. *radiata* must suffer very severe needle cast before it is outgrown by *P*. *muricata* (Ades et al., 1992; Burdon & Low, 2020). In the northern populations of *P*. *muricata*, however, selective pressures imposed by such foliage pathogens may have favored winter dormancy. Winter cold in their coastal environments seems unlikely to be a strong selective force, especially as *P*. *radiata* (of origin 36^1^/_2_−37°N) is notable for growing season frost tolerance (ca. −6°C). This tolerance exceeds that of material derived from one such *P*. *muricata* population (Lat. 39−39^1^/_2_°N) (Menzies & Holden, 1981).

##### Western gall rust

A prominent shoot pathogen affecting pines within and beyond the natural ranges of *P*. *radiata* and *P*. *muricata* is *Endocronartium harknessii* (J. P. Moore) Hirats., which causes western gall rust (Old, 1981; Ramsfield et al., 2007). It infects soft, elongating shoots during spring to early summer. In certain years, when cool, moist conditions persist during the infection season, abundant “wave year” infection can occur. We suggest that the late flushing in the northern populations of *P*. *muricata*, while it may reduce growth potential, reduces exposure to infection hazard in a trade‐off. By comparison, *P*. *radiata*, with its earlier shoot flushing, is vulnerable to infection over a longer season, although its dryer habitats, all south of San Francisco Bay, mean that infection hazard is lower. The fitness advantage of the greater growth potential of *P*. *radiata* resulting from the longer growing season, maybe with some enhanced genetic resistance, evidently outweighs the fitness cost of the associated extension of the infection season in its habitat. The fitness cost of susceptibility is likely mitigated by susceptibility being largely confined to young trees (Old et al., 1986) which often arise at high density from stand‐replacing fires. Selection for resistance, with gall rust infections affecting competitive ability, thereby contributing to the natural self‐thinning, would represent essentially “soft” (density‐dependent) selection.

##### Discussion

There is thus reason to suspect that both foliage pathogens and western gall rust have imposed selective pressures contributing to the comparative seasonal phenology of *P*. *radiata* and northern populations of *P*. *muricata*. Problematically, however, this interpretation does not account for the flushing and pollination dates of the southern populations of *P*. *muricata* (<37°N). Brown (1966), recording pollination dates, which are also a reliable proxy for flushing dates, observed later pollination than in these populations than in *P*. *radiata*, although those populations flush earlier than the northern populations. These populations are also acutely susceptible to foliage disease (Ades et al., 1992; Burdon & Low, 2020) but are naturally exposed to low disease hazard. Brown's observations were made in an essentially common garden situation near Canberra, Australia. There is only one location, Monterey, where the two species naturally co‐occur, and different pollination dates are putatively an adaptive crossing barrier there. Later pollination than in *P*. *radiata* in other southern populations of *P*. *muricata*, however, is not so readily explained, although it did not closely fit a clinal pattern. Moreover, the coolness of the trial site, near Canberra, may have distorted the comparative phenology of the southern populations.

#### 2.3.4 *Quercus* *petraea*

Flushing date can also involve a trade‐off in defenses between climatic and biotic factors. An example has been documented for sessile oak (*Quercus petraea* (Matt.) Liebl.) in the Pyrenees mountains (Dantec et al., 2015; Desprez‐Loustau et al., 2010). The abiotic factor is late spring frosts, and the biotic factor is oak powdery mildew (caused by *Erysiphe quercicola* Takam et al.). Late flushing is a defense against the frosts, whereas early flushing helps protect against the mildew. At higher altitudes, where late frost represents the main adaptive hazard, late‐flushing genotypes are favored. At lower altitudes, which are more conducive to the powdery mildew but less subject to late frosts, early flushing genotypes are favored. Indeed, the greatest incidence and severity of disease occur at intermediate altitudes. With the trade‐off meaning no closely defined or geographically broad optimum for flushing date, the large tree‐to‐tree variability in flushing date, especially at lower altitudes (Alberto et al., 2011), is not surprising. Some validation of the interpretation could in principle be obtained by studying the impact of chemical control of the pathogen, but such a measure faces prohibitive practical difficulties.

#### 2.3.5 Pathogen‐induced perturbations of phenology

In addition to putative cases of pathogens operating as selective influences on natural seasonal phenology, pathogens can exert more direct phenotypic effects on features of the underlying seasonal phenology of the hosts. Classically, foliage infection is a widespread cause of premature leaf fall. Such cases generally entail hosts responding to infection by abscission of foliage or foliage structures. The responses can be adaptively significant, as defense mechanisms for the hosts (Fraser et al., 2016). In some cases, shedding infected foliage may remove inoculum, as well as possibly denying a pathogen further sustenance. This appears to be so in the case (already mentioned) of needle cast associated with *Cyclaneusma minus* in pine species. In some other cases, prompt shedding of foliage can prevent dangerous spread of the pathogen within the host. With white pine blister rust, caused by *Cronartium ribicola* J. C. Fisch., prompt abscission of infected fascicles can serve as an effective resistance mechanism in both *Pinus monticola* Douglas *ex* D. Don (Hoff & McDonald, 1971) and *P*. *armandii* Franch (Hoff & McDonald, 1975). The effect of a plant–pathogen interaction at the level of a plant may differ from the impact on individual infected tissues; for example, powdery mildew infection of pedunculate oak (*Q*. *robur*) leaves resulted in earlier senescence, but higher average infection promoted later autumn phenology in those seedlings overall (Mutz et al., 2021). While infection‐related foliage abscission typically shows seasonality governed by infection and/or sporulation cycles of the pathogens, along with seasonal fluctuations in the host's carbon economy, this does not mean that the abscission is intrinsic to the host's seasonal phenology.

In contrast to infection‐triggered foliage abscission, infection of larch (*Larix*) foliage by *Hypodermella laricis* Tuboef can stop natural abscission, thereby keeping the foliage as a continuing source of inoculum (Cohen, 1987).

### 2.4 Herbivory as a putative driver

#### 2.4.1 Woody perennials

It has been noted that the timing synchronization of seasonal flushing can represent a balance between the advantages of flushing as soon as conditions favor growth and the safety of delaying it until after almost all threat from climatic damage. In temperate zones, where seasons are defined primarily by temperatures, the classic danger is late (spring) frost. However, it is unclear whether close synchronization of flushing is necessarily an adaptive consequence of meeting that balance. An alternative interpretation is that close synchronization of bud burst and shoot and leaf extension means a brief period of “predator satiation” or “predator swamping” (cf. Emlen, 1966; Molles, 2002). This would represent the period of maximum food value for browsers or other herbivores, after which the food value declines sharply. That decline, if it helps to limit the herbivore carrying capacity of an ecosystem, also represents a feedback mechanism to reduce browsing pressure, which, in turn, may reduce the ecological importance of other defenses against herbivory. A question arising with deciduous woody perennials is whether, or how much, any such predator satiation defense may be incidental to the advantage of quick and complete seasonal restoration of photosynthetic capacity. The *Quercus petraea*/powdery mildew case, with tree‐to‐tree variation in flushing date, suggests that it is not entirely so.

With evergreens, in which complete seasonal restoration of photosynthetic capacity is not an issue, the expected premium for earliness of flushing is less. In this connection, while apparently not documented, the very brief flushing periods of some conifers, notably some firs (*Abies* spp.} in very mesic habitats, strongly suggests a predator satiation strategy. Proving the postulate, however, is far more difficult than proposing. But there would be a scope for documenting the phenology in relation to climate and moisture status, which could provide evidence against a simple climatic explanation.

Synchronization of flowering has its own advantages, for efficiency of pollination, but it can also create a brief superabundance for herbivores. This could be another “predator satiation” situation (cf. Emlen, 1966; Molles, 2002), but Ims (1990) concluded that for this to operate the reproductive synchrony needs to spread across subpopulation units. Similarly, synchrony may militate against a build‐up of pathogens that specialize on reproductive structures.

For the tropics, where seasonal effects are often dominated by wet or dry seasons rather than temperatures, the flushing behavior can be more complicated than in temperate regions. Therefore, the likely role of herbivory as a phenotypic driver has attracted more attention. Lamarre et al. (2014) report a study, and cite a number of others, of “synchrony of leaf production” in relation to herbivory. The situation represents widespread operation of predator satiation within the tropics. The story, however, can be more complicated. Aide (1992) documents a case of a woody shrub flushing outside the wet season, at a time when herbivore density is low, in “herbivore escape.” But that species also has brief and intensive flushing and flowering, which is consistent with an element of predator satiation. One may suggest that in herbivore escape the species benefits from an outlier status. Such a status can occur among the typically numerous woody species of tropical forests. In this connection, Bagchi et al. (2014) have documented how “negative density dependent” selection pressures from pathogens can tend to favor high species diversity.

Table 2 summarizes, very broadly, inferred levels of alignment between predator satiation and optimization of growth/competitive ability in temperate and tropical zones.

**TABLE 2:** Inferred levels of alignment between predator satiation and optimization of growth potential or competitive ability (see *Woody perennials*)

Factor/Plant category	Temperate zone	Tropics
Main determinant of seasons	Temperature	Rainfall
Plant category		
Deciduous	Very close	Limited
Evergreens	Less close	Limited

#### 2.4.2 Non‐woody plants

Outside woody perennials, three cases are adduced for herbaceous plants, two for pasture plants and one for a collection of species in a Mediterranean climate. In one pasture plant case, the seasonal phenology, seemingly driven by climate, may actually be driven largely by biotic factors in conjunction with climate. Key aspects of pasture plant phenology are seasonality of forage growth and flowering. An extended growing season, meaning extended forage production, is widely sought by plant breeders. However, there is classically a trade‐off between a long growing season and the persistence that avoids a need for frequent sowings to ensure sward renewal.

##### Medicago

Cold tolerance is typically associated, among populations, with level of winter dormancy, suggesting that active winter growth is physiologically incompatible with cold tolerance. Yet, Daday (1964) observed otherwise. He studied geographic races of *Medicago sativa* L. (lucerne or alfalfa). Among the races, he found the expected picture of a close association between level of winter dormancy and cold tolerance, admittedly not with severe winters. But, in the F2 and F3 generations of an interpopulation diallel cross that association broke down. This independence of inheritance of the two traits conflicted strongly with accepted doctrine, yet no attempt to ascertain its underlying evolutionary significance appears to have been published. We postulate that the observed winter dormancy was in large measure a defense against browsing pressure, rather than being a directly climatic adaptation. Grazing pressure would be intense in winter, and very depleting of the plants’ resources if they were making active growth, presumably leading to selection for winter dormancy. With severe winters, however, dormancy would likely be physiologically obligate, but that would not preclude breeding to extend autumn growth (Castonguay et al., 2006). Our hypothesis could be experimentally probed by studying the effect of winter grazing pressure on persistence of plants that maintained varying levels of winter growth; with suitable material available that should be readily feasible. Assembling suitable material might be a challenge, and an area of ground would need to be available. Actual grazing would require livestock, but grazing might be simulated.

##### Pasture grasses

In the other case involving pasture plants, namely forage grasses, a biotic driver of phenology has been long recognized, at least implicitly. A key phenological trait is the heading season, involving the seasonal timing and duration of seed head development.

Late and brief heading and a semi‐prostrate habit are features of persistent “pasture type” cultivar strains, bred from material with an ancestral history of adaptation to intense grazing (e.g., Charles, 1961). By contrast, “hay type” strains do not have the same history of adaptation to grazing. They have earlier and longer heading seasons and a more erect habit, and much less sward persistence.

##### Other plants

Much more recently, Waterton and Cleland (2016) studied six local and six non‐local plant species grown in a common Californian Mediterranean climate where two species of rabbit applied herbivory pressure. In the absence of the herbivores, early seasonal commencement of growth (shown by the non‐local species) conferred a productivity advantage, but with herbivory delayed commencement was associated with an advantage. Thus, a delay was evidently a defense against herbivory, in a classic trade‐off between defenses and growth potential (cf. Loehle & Namkoong, 1987; Strauss et al., 2002).

## 3 ONTOGENETIC PHENOLOGY

Apart from the progression with size and/or age to reproductive competence, the process of maturation can entail radical differences between juvenile and adult habits, in a phenomenon called heteroblasty. This is common and often very pronounced in the New Zealand flora (Greenwood et al., 1977). Elsewhere, less extreme differences between juvenile and adult habits are generally the norm for woody perennials. Even vegetatively, maturation can bring some changes in seasonal phenology, very often in a progressively stronger expression of the seasonal phenology (Burdon, 1994; Norskov‐Lauritsen, 1963; Wareing, 1958).

### 3.1 New Zealand flora

The heteroblasty in various New Zealand trees and shrubs has been postulated as a defense against vertebrate herbivory (Greenwood et al., 1977). Specifically, various such plant species have a juvenile habit with a tangle of thin, wiry stems bearing very small leaves. This habit is seen as being unrewarding for the large, flightless birds that were the ground‐dwelling browsers, or else resilient to browsing damage. Despite controversy, and difficulty of proof because those browsers are extinct, definite support for the postulate has been obtained. In a neighboring island flora, with closely related taxa but historically lacking those birds, there is not the same heteroblasty (Burns et al., 2009; Greenwood, 1992). Other, although less striking, cases of heteroblasty conferring defenses or resilience against ground‐dwelling herbivores include the production of sharp spines on foliage or stems during a juvenile phase. Seedlings of the apple tree are a well‐known example of spines being confined to the juvenile phase.

### 3.2 Two pines

In the main landmasses, mammals are typically the main ground‐dwelling browsers. While extreme heteroblasty is seldom evident, as it is in New Zealand, there are cases where ontogenetic phenology can be interpreted in terms of changes in both palatability and browse resilience. *Pinus radiata* is an example. In it, the juvenile to adolescent habit is bushier and more intensively branched than the adult habit (Burdon et al., 2017), and resilience to browsing is high. The adult habit, however, is associated with greater palatability to ground‐dwelling browsers and less resilience. This is notoriously evident in adult‐phase scions grafted for seed orchards onto small seedlings, the scions attracting far more deer browsing than seedlings. There is evidently a trade‐off of resistance or resilience to browsing against pathogen tolerance. Juvenile material is more susceptible to some pathogens, notably *Endocronartium harknessii* (Moore) Hirats., which causes western gall rust (Old et al., 1986), and *Dothistroma septosporum* (Dorog.) M. Morelet (syn. *D*. *pini* Hulbary) (Burdon et al., 2017). Losses of some individual seedlings to pathogens, directly or through impaired competitive ability in the case of western gall rust, as part of density‐dependent selection, may not impair population fitness in the thickets of regeneration that often occur naturally. However, another advantage of a juvenile phase can lie in how strongly a longer growing season (cf. Norskov‐Lauritsen, 1963) can contribute to growth potential while tree growth is still partly exponential. At least one other pine, *P*. *taeda* L., shares the feature of being susceptible to an important gall rust pathogen, *Cronartium fusiforme* Hedgcock et Hunt ex Cummins, in young trees rather than later (Powers et al., 1981).

### 3.3 *Eucalyptus* *nitens*

While only incidentally reported as such, *Eucalyptus nitens* (H. Deane et Maiden) Maiden is a case involving a combination of strong ontogenetic phenology and both herbivory and pathogen attack. This species has major geographic (provenance) variation in the duration of juvenile foliage production (Pederick, 1979). The adult phase is subject to insect herbivory, while the juvenile phase is very subject to a foliage disease (Johnson & Wilcox, 1989). Provenance variation evidently reflects the comparative selective pressures imposed by insect pests and pathogens in respective habitats. Thus, provenances with persistent juvenile characteristics, while enjoying longer protection against insect herbivory, are vulnerable to foliage disease with warm, humid summers. Conversely, those provenances with a brief juvenile phase, while less vulnerable to foliage disease, are more so to insect herbivory.

## 4 INDIVIDUAL AND POPULATION FITNESS AND SOME RAMIFICATIONS

Relationships between individual and population fitness in this connection can be problematic. Population fitness depends not just on the metric values for the fitness traits in its habitat which determine individual fitness but also on the amount of heritable variation for those traits. In the poplar case, key adaptive traits in the wild would include pathogen resistance, timing of seasonal growth, and the sexual reproductive potential that allows genetic selection to operate. Thus, the impact of some ill‐adapted segregants can be outweighed by a concurrent incidence of segregants that excel their parents’ fitness, since the latter contribute most to the parentage of the next generation. However, with pathogens involved, population fitness can be complicated by the nature of infection processes. With a “polycylic” pathogen, there can be both reinfection from within the host individual and cross‐infection between individuals. For instance, where pathogens impair fitness, an individual becoming deciduous gains little or no protection if the population remains evergreen and can thereby provide a continuous supply of inoculum. In this way, becoming deciduous is unlikely to be advantageous, unless either this results in asynchrony between the window of host susceptibility and presence of inoculum or individuals are too sparse to favor cross‐infection. An individual becoming evergreen, however, might impair the fitness of the rest of the population through producing a continuous supply of inoculum, unless it suffers so badly from internal reinfection as to incur selective elimination. To meet that condition, internal reinfection would presumably need to predominate strongly over cross‐infection between individuals, which may be common with polycyclic infection.

Becoming evergreen could require evolution of enhanced pathogen resistance and/or some more direct fitness advantage of the habit. Natural selection for resistance may be limited by “selective opportunity cost” whereby the fitness gains from additional resistance are in trade‐off with the fitness gains from responses to other selective pressures. Some of those gains, it may be noted, will be needed to make good the decay of non‐additive genetic components of fitness that occurs upon sexual reproduction. Given the dynamic nature of biotic interactions, along with pathogen mutations, natural selection for ecological fitness can be expected to entail a “red queen” model (Van Valen, 1973) requiring endless genetic shifts to maintain fitness. With long‐lived perennials, however, such shifts may occur most readily within generational cohorts, during lifespans. With the complex and highly dynamic poplar rust pathosystem, poplar plantations have proved notoriously vulnerable to pathotype mutations (e.g., Persoons et al., 2014). Such plantations are typically monoclonal, which makes them convenient to produce and manage. Present understanding of pathosystems suggests that natural populations of poplars, reproducing by seed, might be expected to be more resilient toward the rusts. However, fully functioning natural populations, which would provide a good basis for comparison, are becoming increasingly scarce.

It must be noted that the biotic vulnerability of poplar plantations, based on species that are *Melampsora* hosts, contrasts with the biotic resilience of natural stands of American (or trembling) aspen (*Populus tremuloides* Michaux) which are typically monoclonal (Latutrie et al., 2015). The aspen, with different pathogens, is evidently involved in very different pathosystems.

## 5 PREADAPTIVE RELATIONSHIPS?

As indicated above, seasonal timing of phenological events may serve as defense against pathogens through avoidance of infection seasons while the plant is vulnerable. Alternatively, the timing may be a source of vulnerability. This seems evident in some cases of freshly introduced pathogens, rather than in historically co‐evolved cases. With Dutch elm disease, caused by ascomycete fungi *Ophiostoma* spp., early bud burst and flushing, while possibly incurring climatic vulnerability, can evidently avoid the height of the infection season (Ghelardini & Santini, 2009). With sudden oak death, caused by *Phytophthora ramorum* Werres et al., host vulnerability has been linked to timing of bud burst and onset of cambial activity in the hosts (Dodd et al., 2008). Similarly, the pathogen *Austropuccinia psidii* (G. Winter) Beenken, a rust affecting a wide range of hosts in the Myrtaceae that has invaded parts of Asia and the Pacific, can only infect actively flushing host tissues (Beresford et al., 2020). In each case, invasive pathogens appear likely to place strong selective pressures on the phenology of native host populations, and understanding the potential for adaptive responses appears to be worth further study.

## 6 CONCLUDING

We propose that pathogens can be strong evolutionary drivers of seasonal phenology, especially in respect of deciduousness. This appears to be largely novel. Some evidence is given for our proposal for deciduousness in a temperate zone. In the tropics, the proposal is made for want of any other satisfactory explanation. Other examples are given of how pathogens may influence seasonality of shoot flushing, where simple climatic explanations seem inadequate. We suggest that evolutionary barriers can operate against a shift from a deciduous to an evergreen habit in some temperate zone cases.

For herbivory, we have proposed that predator satiation may operate as an evolutionary driver in addition to purely climatic adaptation to the occurrence of winters. Herbivory is also proposed as an evolutionary constraint on growing or flowering seasons. For the tropics, where climates are largely characterized by wet and dry seasons, predator satiation has already been implicitly identified as a quite common evolutionary driver of seasonal phenology.

Ontogenetic phenology appears strongly influenced evolutionarily by biotic factors, including both herbivory and pathogens.

How a particular situation plays out will depend on many factors, notably the general climatic context and the constraints it imposes (along with other environmental factors), and the nature of biotic pressures, namely pathogens and/or herbivory. More specifically, the factors for pathogens involve the reproductive biology of pathogens including seasonality; the host plant ecology, in the scale from highly gregarious to essentially solitary; and the genetic systems of both host and pathogens. For herbivory, they include the life cycles and seasonal abundance of insects, along with the nature and abundance of vertebrate browsers. Not to be ignored are interactions with other plants in the ecosystem and among herbivores.

Influences of climate are both direct and indirect, the latter category involving how much climate favors pathogens and herbivores. With pathogens, there is the classic “triangle” of interactions involving host, pathogen, and the environment, so the direct and indirect evolutionary influences are in some degree interdependent. With the poplar/*Melampsora* pathosystem, a direct influence of a climate that is milder than the native one may favor an evergreen habit. But the indirect influence, through the effect of the same climate on the pathogen, is likely to militate against that shift. Often, however, the evolutionary pressures from climatic and biotic factors would be mutually reinforcing, as in the *Medicago* case.

In calling for a broad evaluation of biotic factors as evolutionary drivers (or co‐drivers) of plant phenology, the coverage is preliminary and selective, representing an alert to the topic. Even a more systematic literature review would still require interpretation of individual cases. Challenges certainly exist in such evaluation. To identify likely cases of biotic drivers, we consider it appropriate to look largely to those who are closely familiar with individual plant species and their ecology. Then, detailed review and analysis of available evidence is needed, but interpretation in individual cases may still be problematic. From there, the general postulate of biotic influences will need to be carried forward into hypotheses that are observationally or experimentally testable. While woody perennials have provided tantalizing case histories, they can also pose major logistical challenges in testing hypotheses.

## AUTHOR CONTRIBUTIONS

**Rowland D. Burdon:** Conceptualization (lead); Investigation (lead); Methodology (equal); Visualization (supporting); Writing – original draft (lead); Writing – review & editing (equal). **Michael J. Bartlett:** Conceptualization (supporting); Funding acquisition (lead); Methodology (equal); Resources (lead); Visualization (lead); Writing – original draft (supporting); Writing – review & editing (equal).

## CONFLICT OF INTEREST

None declared.

### OPEN RESEARCH BADGES

None.

## Data Availability

Not applicable as no fresh or hitherto unpublished data are involved.
